# 

**Published:** 1892-03

**Authors:** 


### BOOKS AND PAMPHLETS RECEIVED.

Transactions of the American Gynecological Society. Volume XVI. For the year 1891. Philadelphia: Wm. J. Dornan, printer. 1891.

The Hydriatic Treatment of Typhoid Fever, according to Brand, Tripier, and Bouveret, and Volg. By Chr. Sihler, M. D., Ph. D., Professor of Histology in the Medical Department of the Western Reserve University, former Fellow of the Johns Hopkins University, and Assistant in the Biological Laboratory. Published by Chr. Sihler, 832 Scranton avenue, Cleveland, Ohio.

Essentials of Physics, arranged in the form of questions and answers. Prepared especially for Students of Medicine. By Fred J. Brockway, M. D., Assistant Demonstrator of Anatomy at the College of Physicians and Surgeons, New York. With 155 illustrations. Philadelphia: W. B. Saunders, 913 Walnut street. 1892.

The Chinese, their Present and Future: Medical, Political, and Social. By Robert Coltman, Jr., M. D., Surgeon-in-Charge of the Presbyterian Hospital, at Teng Chow Fu; Consulting Physician of the American Southern Baptist Mission Society; Examiner in Surgery and Diseases of the Eye for the Shantung Medical Class; Consulting Physician to the English Baptist Mission, etc. Illustrated with fifteen fine photo-engravings. Philadelphia and London: F. A. Davis, publisher, 1891.

Stricture of the Rectum: A study of one hundred and thirty-eight cases. Second edition, enlarged. By Charles B. Kelsey, M. D., New York, Professor of Diseases of the Rectum at the New York Post-Graduate School and Hospital; late Professor of Rectal Surgery at the University of Vermont, etc., etc.

The Principles of Bacteriology: A Practical Manual for Students and Physicians. By A. C. Abbott, M. D., First Assistant, Laboratory of Hygiene, University of Pennsylvania, Philadelphia. With illustrations. Philadelphia: Lea Brothers & Co. 1892.

Addresses, Papers and Discussions in the Section of State Medicine, at the Forty-second Annual Meeting of the American Medical Association, at Washington, D. C., May 5-8, 1891. Chicago: Printed at the office of the Association. 1891.

Consumption: How to Prevent It and How to Live with It. Its Nature, its Causes, its Prevention, and the Mode of Life, Climate, Exercise, Food, Clothing, necessary for its cure. By N. S. Davis, Jr., A. M., M. D., Professor of the Principles and Practice of Medicine, Chicago Medical College; Physician to Mercy Hospital; Member of the American Medical Association, Illinois State Medical Society, Chicago Medical Society, Chicago Academy of Sciences, Illinois State Microscopical Society; Fellow of the American Academy of Medicine; author of a hand-book on Diseases of the Lungs, Heart and Kidneys. Philadelphia and London: F. A. Davis, publisher. 1891.

Syphilis in Ancient and Prehistoric Times. By Dr. F. Buret, Paris, France. Translated from the French with notes, by A. H. Ohmann-Dumesnil, M. D.. Professor of Dermatology and Syphilology in the St. Louis College of Physicians and Surgeons; Consulting Dermatologist to the St. Louis City Hospital; Physician for Cutaneous Diseases to the

Alexian Brothers' Hospital; Dermatologist to St. Margaret's Hospital, etc., etc. Syphilis Today and Among the Ancients; in three volumes. Volume I., *Nihil sub sole novum.* Philadelphia and London: F. A. Davis, publisher. 1891.

Disease of the Bladder and Prostate. By Hal. C. Wyman, M. Sc., M. D., Professor of Surgery in the Michigan College of Medicine and Surgery, Detroit; Member of the American Medical Association, Michigan State Medical Society, Michigan Surgical and Pathological Society, Detroit Academy of Medicine; Surgeon Detroit Emergency Hospital, etc., etc. George S. Davis, Detroit, Mich. 1891.

Sleep, Insomnia, and Hypnotics. By E. P. Hurd, M. D., Member of the Massachusetts Medical Society; Member of the Climatological Society; Member of the Société de Medicine Pratique (Paris, France); one of the Physicians in the Anna Jaques Hospital, Newburyport, Mass. George S. Davis, Detroit, Mich. 1891.

A Manual of Operative Surgery. By Frederick Treves, F. R. C. S., Surgeon to, and Lecturer on Anatomy at the London Hospital. In two octavo volumes, containing 1550 pages, with 422 illustrations, mostly original. Per set, cloth, $9.00; leather, $11.00. Philadelphia: Lea Brothers & Co. 1892.

Surgical Diseases of the Ovaries and Fallopian Tubes, including Tubal Pregnancy. By J. Bland Sutton, F. R. C. S., Assistant Surgeon to the Middlesex Hospital, London; late Hunterian Professor, Royal College of Surgeons, of England. In one 12mo volume of 514 pages, with 119 engravings and five colored plates. Cloth, $3.00. Philadelphia: Lea Brothers & Co. 1892.

A Text-Book of Chemical Physiology and Pathology. By W. D. Halliburton, M. D., B. Sc., M. R. C. P., Professor of Physiology at King's College, London; Lecturer on Physiology at the London School of Medicine for Women; late Assistant Professor of Physiology at University College, London. With 104 illustrations. London: Longmans, Green & Co. New York: 15 East Sixteenth street. 1891.

Essentials of Medical Electricity. By D. D. Stewart, M. D., Demonstrator of Diseases of the Nervous System, and Chief of the Neurological Clinic, in the Jefferson Medical College; Physician to St. Mary's Hospital and to St. Christopher's Hospital for Children; Fellow of the College of Physicians of Philadelphia, etc.; and E. S. Lawrance, M. D., Chief of the Electrical Clinic and Assistant Demonstrator of Diseases of the Nervous System in the Jefferson Medical College; Physician to the Dispensary of St. Christopher's Hospital for Children, etc. With sixty-five illustrations. Philadelphia: W. B. Saunders, 913 Walnut street. 1892.

First Lines in Midwifery: A Guide to Attendance on Natural Labor for Medical Students and Midwives. By G. Ernest Herman, M. D., F. R. C. P., Obstetric Physician to the London Hospital, and Lecturer on Midwifery; Examiner in Midwifery to the Royal College of Surgeons. In one 12mo volume of 198 pages, with eighty illustrations. Cloth, $1.25. Students' Series of Manuals. Philadelphia: Lea Brothers & Co. 1892.

A Dictionary of Treatment, or Therapeutic Index, including Medical and Surgical Therapeutics. By William Whitla, M. D., Professor of Materia Medica and Therapeutics in the Queen's College, Belfast. Revised and adapted to the Pharmacopeia of the United States. In one octavo volume of 917 pages. Cloth, $4.00. Philadelphia: Lea Brothers & Co. 1892.

Notice to Contributors.—We are glad to receive contributions from every one who knows anything of interest to the profession. Articles designed for publication in the Journal should be handed in before the first day of the month. The Editors are not responsible for the views or opinions of contributors. All communications should be addressed to the Managing Editor, 284 Franklin St., Buffalo, N. Y.

